# Growth hormone and melatonin prevent age-related alteration in apoptosis processes in the dentate gyrus of male rats

**DOI:** 10.1007/s10522-013-9443-6

**Published:** 2013-07-13

**Authors:** R. A. Kireev, E. Vara, J. A. F. Tresguerres

**Affiliations:** 1 Department Physiology, Medical School, University Complutense of Madrid, Madrid, Spain; 2 Department Biochemistry and Molecular Biology, Medical School, University Complutense of Madrid, Madrid, Spain; 3 Laboratory of Experimental Endocrinology, Department of Physiology, School of Medicine, Complutense University, Avda Camon y Cajal s/n., 28040 Madrid, Spain

**Keywords:** Aging, Dentate gyrus, Apoptosis, Sirtuins, Growth hormone, Melatonin

## Abstract

It has been suggested that the age-related decrease in the number of neurons in the hippocampus that leads to alterations in brain function, may be associated with an increase in apoptosis due to the reduced secretion of growth hormone (GH) and/or melatonin in old animals. In order to investigate this possibility, male Wistar rats of 22 months of age were divided into three groups. One group remained untreated and acted as the control group. The second was treated with growth hormone (hGH) for 10 weeks (2 mg/kg/d sc) and the third was subjected to melatonin treatment (1 mg/kg/d) in the drinking water for the same time. A group of 2-months-old male rats was used as young controls. All rats were killed by decapitation at more than 24 month of age and dentate gyri of the hippocampi were collected. Aging in the dentate gyrus was associated with an increase in apoptosis promoting markers (Bax, Bad and AIF) and with the reduction of some anti-apoptotic ones (XIAP, NIAP, Mcl-1). Expressions of sirtuin 1 and 2 (SIRT1 and 2) as well as levels of HSP 70 were decreased in the dentate gyrus of old rats. GH treatment was able to reduce the pro/anti-apoptotic ratio to levels observed in young animals and also to increase SIRT2. Melatonin reduced also expression of pro-apoptotic genes and proteins (Bax, Bad and AIF), and increased levels of myeloid cell leukemia-1 proteins and SIRT1. Both treatments were able to reduce apoptosis and to enhance survival markers in this part of the hippocampus.

## Introduction

The hippocampus is a target of age-related physiological and structural changes. Alterations in the hippocampus during aging are paralleled by behavioral and functional deficits in hippocampus-dependent learning and memory tasks (Rosenzweig and Barnes 2003). Recent microarray studies of hippocampal gene expression, identified several aging-dependent upregulated processes, including lipid catabolism, proteolysis, cholesterol transport, and myelinogenesis in normally aging rats (Rowe et al. 2007; Kadish et al. 2009)_._

In humans, apoptosis occurs in the developing nervous system to eliminate neurons with erroneous or inadequate projections (Chan et al. 2002), whereas enhanced apoptosis in the adult brain is also characteristic of several pathologies of the central nervous system (CNS) (Mattson 2006; Kim and Sun 2011). It has been suggested that the decrease in the number of neurons by apoptosis may be associated with trophic factor(s) deprivation (Ambacher et al. 2012) or with the absence or reduction of anti-apoptotic proteins such as B-cell lymphoma 2 (Bcl-2) (Allsopp et al. 1993). Many apoptosis-related factors have been demonstrated to be up-regulated in the immature brain, such as caspase-3, Apaf-1 and Bcl-2-associated X protein (Bax) (Ota et al. 2002; Troy et al. 2011). Inhibitor of apoptosis (IAP) family of proteins like neuronal inhibitor apoptosis protein (NIAP), X-linked inhibitor of apoptosis protein (XIAP), and cIAP-2 play also a role in the regulation of neuronal death and in several models of disease. NIAP directly inhibits caspase-3, -7, and -9 and XIAP directly inhibits also caspase-3 and caspase-7 (Prunell and Troy 2004).

Sirtuin1 (SIRT1) is a mammalian nicotinamide adenine dinucleotide (NAD)-dependent histone deacetylase (HDAC) that down-regulates the acetylation levels of many regulatory proteins involved in energy homeostasis, DNA repair, cell survival, and lifespan extension (Kwon and Ott 2008). SIRT1 has been shown to be associated with reduced apoptosis, whereas inactivation of SIRT1 could on the contrary promote translocation of Bax from the cytosol to mitochondria (Cohen et al. 2004). SIRT1 also protects against amyloid-β toxicity in cell culture and neurodegeneration in the p25/CDK5 mouse model, which recapitulates aspects of Alzheimer’s disease pathology and tauopathy (Kim et al. 2007).

Activity of the growth hormone (GH)/insulin like growth factor (IGF-I) axis undergoes an age-related decline, including reduced spontaneous GH secretion and circulating IGF-I levels, which may reach values that are similar to those found in GH-deficient patients (Ghigo et al. 2000). In both elderly and GH-deficient adults, this decreased GH/IGF-I activity has been associated with changes in body composition and metabolism, altered sleep patterns and reduced cognitive function. GH replacement therapy has been found to improve some age-dependent cognitive functions, such as memory, motivation, or mental processing speed (Nass et al. 2009; Quik et al. 2010). In a dwarf rat model with reduced levels of serum GH and IGF1 beginning before adolescence, early intervention with GH for 10 weeks starting around puberty was reported to ameliorate age-related pathology in later life and increase lifespan (Sonntag et al. 2005). A significant decrease has been observed in the density of GH binding with increasing age (over 60 years old) in the choroid plexus, hypothalamus, hippocampus, pituitary, and putamen (Lai et al. 1993). Progressive loss of activated precursors and neurons has been shown to correlate with an age-dependent decline in GH secretion in both rodents and humans (Nieves-Martinez et al. 2010). Knock-down of GH by gene silencing in cells of this cultured embryonic neural retina cell line, using NR-cGH-1 siRNA, correlates with the increased appearance in the cultures of cells with apoptotic nuclear morphology (Sanders et al. 2010).

Melatonin binding sites do also exist in the hippocampus of several mammals. MT1 and MT2 receptors have been localized in the dentate gyrus, CA3 and CA1 regions and subiculum of the hippocampus (Musshoff et al. 2002). Melatonin production by the pineal is severely restricted in advanced age in humans as well as in experimental animals. Other studies have demonstrated an age-related decrease of 2-[125]-iodomelatonin binding and MT1 mRNA in the CNS of rat and mice (Pandi-Perumal et al. 2008). For instance, Laudon et al. (1988) reported an age-related lowering in the density of melatonin binding sites in discrete rat hypothalamic and hippocampal areas. Many experimental data have demonstrated that, melatonin is an anti-apoptotic mediator. So, melatonin supplementation was able to inhibit apoptosis in amyloid β-peptide (Aβ) injury in hippocampal neurons (Shen et al. 2002) and to suppress NO-induced apoptosis by stimulating of Bcl-2 expression in immortalized pineal PGT-β cells (Yoo et al. 2002).

In a previous publication, we have demonstrated that the number of neurons in the hilus of the dentate gyrus was significantly decreased during aging and that chronic GH treatment was able to prevent this neuronal loss. These findings indicated that GH administration might prevent some of the aging associated with this hippocampal area alteration (Azcoitia et al. 2005). Since no increase in neurogenesis was detected, the maintenance of the neuronal population could be probably due to a reduction of apoptosis. However, previous data supporting this potential pathway were obtained only in whole rat brain (Tresguerres et al. 2008) but not specifically in the denate gyrus.

So, the aim of the present study was to investigate if chronic treatments with exogenous GH and melatonin were able to reduce the increased apoptosis processes observed in the dentate gyrus of old male Wistar rats, leading to a reduction in the total number of neurones in this CNS area after 22 months of age in these animals.

## Materials and methods

### Animals

Male Wistar rats of 2 (6–8 % lifespan) and 22 months (70–75 % lifespan) of age were used. Animals were obtained from Harlan Iberica (Barcelona, Spain) and maintained at a constant temperature (21 ± 2 °C) on a 12-h light/dark cycle and standard laboratory rat chow (A04 Panlab, Barcelona, Spain) with free access to food and water. Rats were treated according to the guidelines of the European Community Council Directives 86/6091 EEC. The average lifespan of Wistar rats in the laboratory was ~34–36 months.

Old animals were divided in three groups at 22 months of age. Untreated control rats (group 1), rats treated with hGH, (group 2) and animals treated with melatonin (group 3). Rats were sacrificed in groups of *n* = 12/13 at more than 24 months of age.

Melatonin (Actafarma, Madrid, Spain) was given in the drinking water at a dose of 1 mg/kg/day for 10 weeks. A fresh melatonin solution was prepared 3 times per week, depending on the water consumption and the weight of the animals to obtain a melatonin dose of 1 mg/kg/day. Water bottles were covered with aluminium foil to protect them from light, and the drinking fluid was changed 3 times weekly. Melatonin was given over 24 h, but we need to take into account that during the day these animals were sleeping, so, normally more than 80 % of the water was drunk during there activity phase, in the night.

Treatment with hGH (Sandoz, Germany) was performed twice daily by sc injections at a dose of 1 mg/kg for 2.5 months (Azcoitia et al. 2005; Tresguerres et al. 2008). We need to take into consideration that the GH used was of human origin, so that the response was not the same as for rat GH. On the other hand, small animals needed a much higher dosage than humans, as was demonstrated by Mordenti and Chappell (1989).

Untreated animals were injected with saline twice daily. A group of 2-months-old animals (*n* = 12) was used as reference control. After the treatment period, animals were sacrificed by decapitation and dentate gyrus samples were immediately dissected. Dentate gyrus was dissected under a binocular as described (Lein et al. 2004; Hagihara et al. 2009), immediately frozen in liquid nitrogen, and stored at −80 °C until use.

### Extraction of tissue samples and determination of HSP 70

Dentate gyrus were quickly dissected and frozen in liquid nitrogen. Frozen organ samples were transferred to 5-ml polypropylene tubes containing 1× Extraction reagent (4 °C) with protease inhibitors (0.1 mM PMSF, 1 μg/ml leupeptin, 1 μg/ml aprotinin, 1 μg/ml pepstatin). Samples were homogenized for 30 s with an electrical homogenizer (Polytron; Brinkmann Instruments, Westminster, NY, USA) and later centrifuged at 21,000×*g* (10 min, 4 °C). The supernatant collected and were stored at −80 °C until assayed for the quantitative presence of HSP 70.

HSP 70 was measured with an ELISA kit according to the manufacturer’s instructions (Assay designs, Stressgen, MI, USA, catalog number: EKS-700B).

A mouse monoclonal antibody specific for inducible HSP 70 is pre-coated on the well of the provided HSP 70 Immunoassay Plate. Inducible HSP 70 is captured by the immobilized antibody and is detected with a HSP 70 specific rabbit polyclonal antibody. The rabbit polyclonal antibody is subsequently bound by a horseradish peroxidase conjugated anti-rabbit IgG secondary antibody. The assay is developed with tetramethylbenzidine (TMB) substrate and blue color develops in proportion to the amount of captured HSP 70. The color development is stopped with acid stop solution. The intensity of the color is measured in microplate reader at 450 nm. HSP 70 concentrations from the sample are quantitated by interpolating absorbance reading from a standard curve generated with the calibrated HSP 70 protein standard provided.

### Western blotting analysis

Western blots were used to measure the protein expression of Bax, Bcl-2-associated death promoter (Bad), myeloid cell leukemia-1 (Mcl-1) and Bcl-2. Briefly, dentate gyrus samples after homogenization with lysis buffer were sonicated, boiled with gel-loading buffer (0.100 M Tris–HCl; 4 % SDS; 20 % glycerol; 0.1 % bromophenol blue) in the ratio 1:1, and protein concentrations were determined by the Bradford methods. Total protein equivalents (25–30 μg) for each sample were separated by SDS-PAGE by using 10 % acrylamide gels and were transferred onto nitrocellulose membrane in a semi-dry transfer system. The membrane was immediately placed into blocking buffer containing 5 % nonfat milk in 20 mM Tris, pH 7.5; 150 mM NaCl; and 0.01 % Tween-20. The blot was allowed to block at 37 °C for 1 h. The membrane was incubated with rabbit polyclonal Bax, Bad, Bcl-2 and with goat polyclonal Mcl-1 (Gene Tex, Inc., CA, USA) (1:1,000) for 2 h at 25–27 °C, followed by incubation in an anti-rabbit or anti-goat IgG-horseradish peroxidase conjugated antibody (1:4,000). After washing with T-TBS, the membranes were incubated with ECL Plus detection reagents (Amersham Life Science Inc., Buckinghamshire, UK), exposed to X-ray film. The films were scanned with densitometer (BioRad GS 800) to determine the relative optical densities. Pre-stained protein markers were used for molecular weight determinations.

### RNA isolation and RT-PCR

RNA was isolated from dentate gyrus samples of male rats using the TRI Reagent Kit (Molecular Research Center, Inc., Cincinnati, OH), following the manufacturer’s protocol. The purity of the RNA was estimated by 1.5 % agarose gel electrophoresis, and RNA concentration was determined by spectrophotometry (260 nm). Reverse transcription of 2 μg RNA for cDNA synthesis was performed using the Reverse Transcription System, (Promega, Madison, WI, USA) and a pd(N)6 random hexamer. RT-PCR was performed in an Applied Biosystems 7300 apparatus using the SYBR Green PCR Master Mix (Applied Biosystems, Warrington, UK) and 300 nM concentrations of specific primers (Table 1). The thermocycling profile conditions used were: 50 °C for 2 m, 95 °C for 10 m, 95 °C for 15 s, 60 °C for 1 m, 95 °C for 15 s, 60 °C for 30 s and 95 °C for 15 s. For the normalization of cDNA loading in the PCR reaction, the amplification the 18S rRNA for every sample was used. Relative changes in gene expression were calculated using the 2-ΔΔCT method.

**Table 1:** Primers used in real-time PCR experiments

Primers		Sequence (5′–3′)
18s	Forward	GGTGCATGGCCGTTCTTA
	Reverse	TCGTTCGTTATCGGAATTAACC
Bcl-2	Forward	CAGGTATGCACCCAGAGTGA
	Reverse	GTCTCTGAAGACGCTGCTCA
BAD	Forward	GCCCTAGGCTTGAGGAAGTC
	Reverse	CAAACTCTGGGATCTGGAACA
BAX	Forward	GTGAGCGGCTGCTTGTCT
	Reverse	GGTCCCGAAGTAGGAGAGGA
XIAP	Forward	GCTTGCAAGAGCTGGATTTT
	Reverse	TGGCTTCCAATCCGTGAG
AIF	Forward	AGTCCTTATTGTGGGCTTATCAAC
	Reverse	TTGGTCTTCTTTAATAGTCTTGTAGGC
NIAP	Forward	GAGAGGTGGCACAGTCAGGT
	Reverse	TAAAACGGCCAGTCCTCAAA
Sirtuin 2	Forward	CCACTGTAACCACGTCTGCTC
	Reverse	CAGTGTCCGAGTCTGAATCCT
Sirtuin 1	Forward	TCGTGGAGACATTTTTAATCAGG
	Reverse	GCTTCATGATGGCAAGTGG
IGF-I	Forward	TGTCGTCTTCACATCTCTTCTACCTG
	Reverse	CCACACACGAACTGAAGAGCGT

18s was used as a housekeeping gene to compare the samples

### Statistical analyses

The results were statistically analyzed with the ANOVA method and a confidence level of 95 % (*p* < 0.05) was considered significant. Results are expressed as the mean ± SEM. Mean comparison was done by the ANOVA analysis of variance followed by a Fisher test.

## Results

**Fig. 1:**
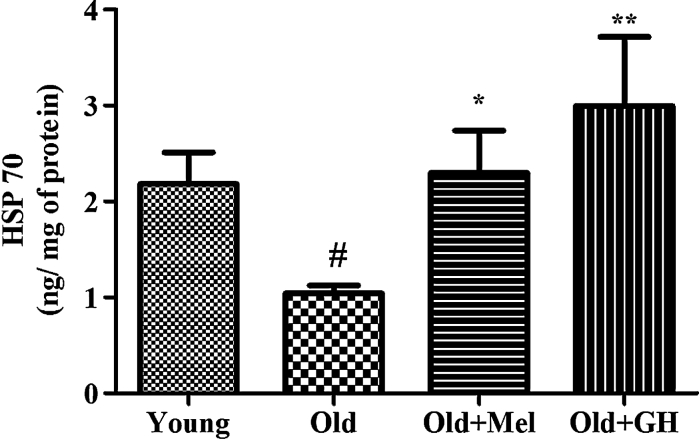
Level of HSP 70 in dentate gyrus of young and old male rats and effect of chronical treatments with melatonin and GH. Data represent mean ± SEM

HSP 70 levels, which are known to prevent apoptosis, were decreased in the dentate gyrus of old male rats as compared to young ones (*p* ≤ 0.03). Administration of GH (*p* ≤ 0.01) and melatonin (*p* ≤ 0.05) significantly elevated the levels of HSP 70 in hippocampus of old rats, with GH showing a more marked effect (Fig. 1).

**Fig. 2:**
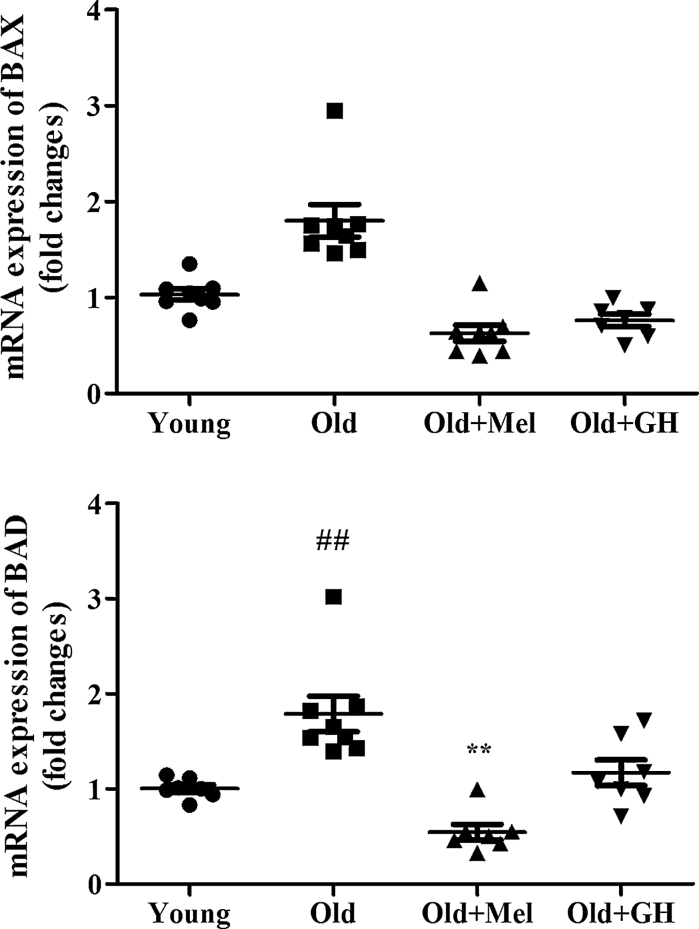
mRNA (*n* = 7/8) expression of BAD and BAX in dentate gyrus of young and old male rats and effect of chronical treatments with melatonin and GH. Data represent mean ± SEM

The gene expressions of pro-apoptotic markers like Bax (*p* ≤ 0.01) and Bad (*p* ≤ 0.05) were increased in the dentate gyrus of old males as compared to young controls (Fig. 2). Melatonin treatment decreased the expression of Bad (*p* ≤ 0.001) and Bax (*p* ≤ 0.001) in dentate gyrus of old animals. However, GH was only able to diminish the mRNA expression of Bax (*p* ≤ 0.001), but not of Bad (Fig. 2).

**Fig. 3:**
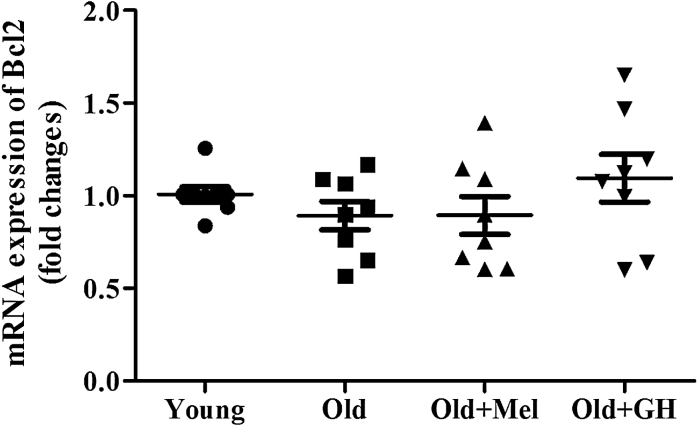
mRNA (*n* = 7/8) expression of Bcl-2 in dentate gyrus of young and old male rats and effect of chronical treatments with melatonin and GH. Data represent mean ± SEM

A slight age-related decrease of Bcl-2 expression in the dentate gyrus was evident in the study, but this trend did not show significant differences. When old rats were treated with GH or melatonin the expression of Bcl-2 did not show significant changes (Fig. 3). However, the Bcl-2/Bax ratio was higher in the group of young animals as compared to old and treatment with GH and melatonin were able to increase this parameter, thus showing that cells in this area of the hippocampus were protected from apoptosis.

**Fig. 4:**
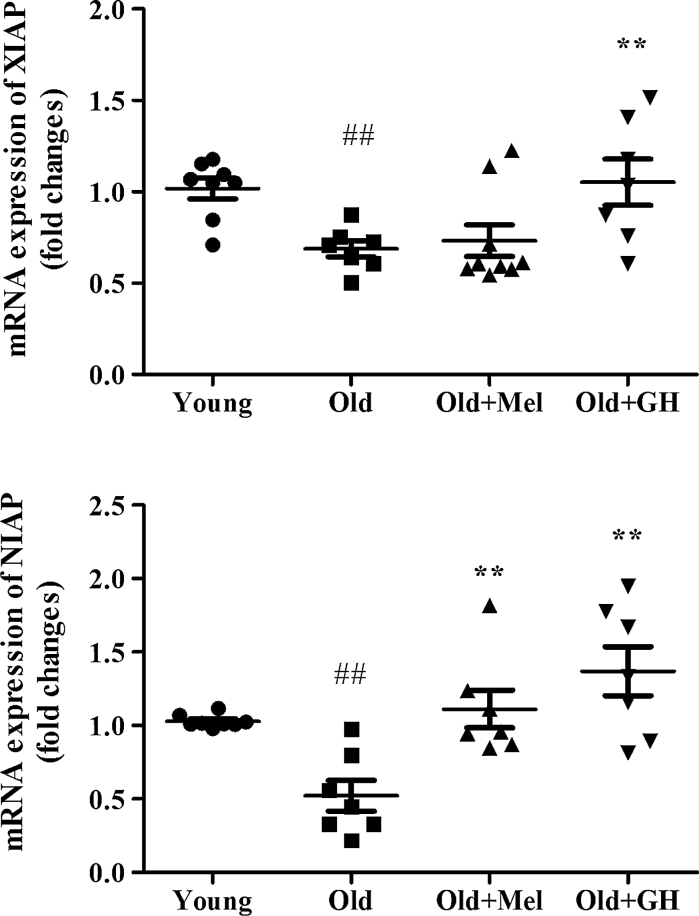
Expression of mRNA XIAP and NIAP in the dentate gyrus of young and old male rats and effect of administration of melatonin and GH. Data represent mean ± SEM (*n* = 7/8)

The mRNA expressions of XIAP (*p* ≤ 0.05) and NIAP (*p* ≤ 0.05) were decreased in dentate gyrus of 24 month old when compared to 2 month old male rats (Fig. 4). When old males were treated with GH, significant increases of gene expression of XIAP (*p* ≤ 0.01) and NIAP (*p* ≤ 0.01) were observed. After chronic treatment with melatonin a significant increase in mRNA expression of NIAP (*p* ≤ 0.01) was detected, whereas XIAP expression did not show any change (Fig. 4).

**Fig. 5:**
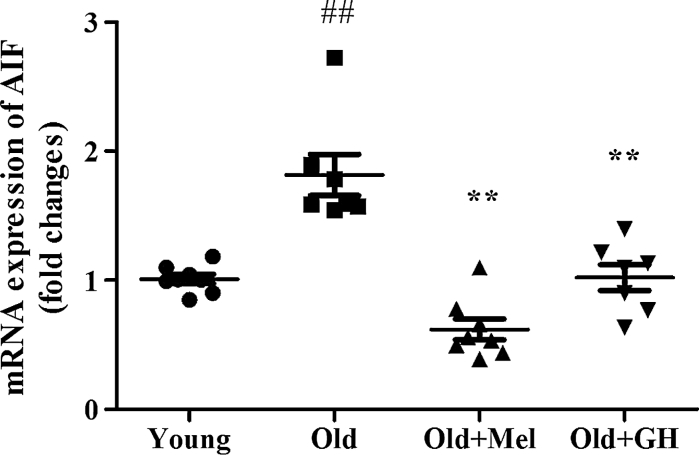
Expression of mRNA AIF in the dentate gyrus of young and old male rats and effect of administration of melatonin and GH. Data represent mean ± SEM (*n* = 7/8)

This hippocampus area of aged rats showed a highly significant increase in mRNA expression of apoptosis-inducing factor (AIF) (*p* ≤ 0.001). Administration of melatonin and GH to old males significantly lowered its mRNA expression (*p* ≤ 0.01) (Fig. 5).

**Fig. 6:**
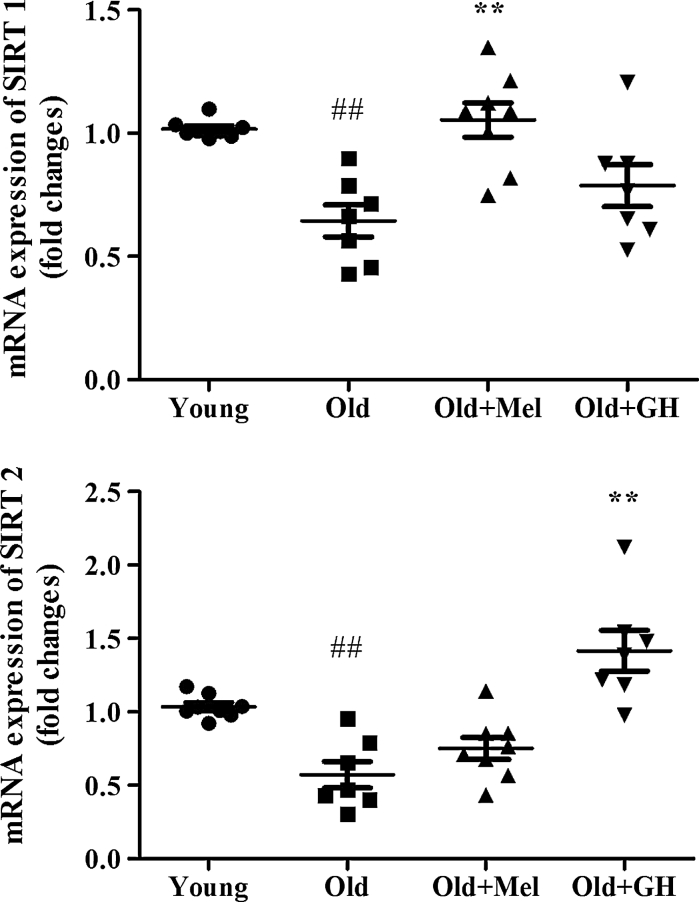
The expression of mRNA SIRT1 and SIRT2 in the dentate gyrus of young and old male rats and effect of administration of melatonin and GH. Data represent mean ± SEM (*n* = 7/8)

Aging induced also a significant decrease in the expression of SIRT1 (*p* ≤ 0.05) and SIRT2 (*p* ≤ 0.05) in the dentate gyrus (Fig. 6). The expression of SIRT2 gene was significantly up-regulated after GH replacement (*p* ≤ 0.05), but melatonin did not produce any effect on this parameter in the group of old male rats (Fig. 6). However, the mRNA expression of SIRT1 was significantly increased in the dentate gyrus of old males, but only after melatonin treatment (*p* ≤ 0.001) (Fig. 6).

**Fig. 7:**
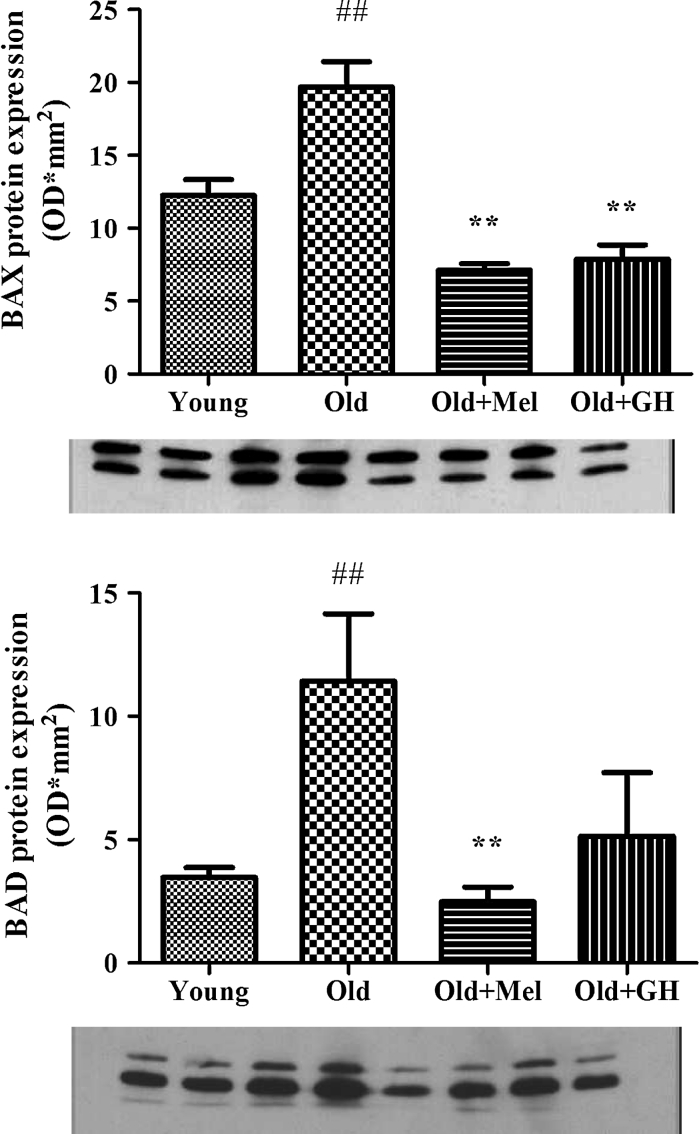
Protein expression of BAD and BAX detected by Western blot in dentate gyrus of young and old male rats and effect of chronical treatments with melatonin and GH. Data represent mean ± SEM (*n* = 4/5)

**Fig. 8:**
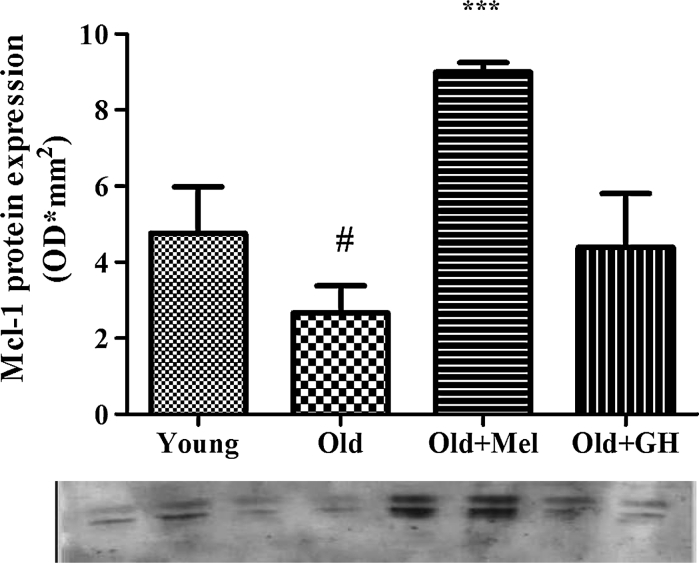
Western blot analysis of Mcl-1 protein expression in the dentate gyrus of young and old male rats and effect of administration of melatonin and GH. Data represent mean ± SEM (*n* = 4/5)

**Fig. 9:**
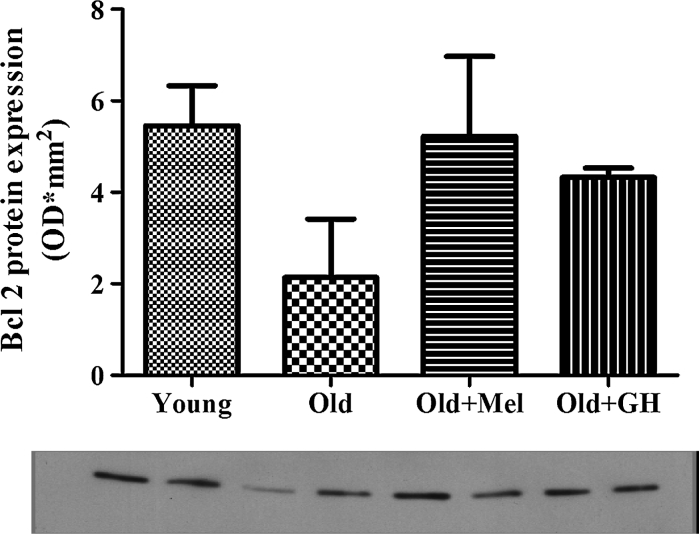
Protein expression of Bcl-2 detected by Western blot in the dentate gyrus of young and old male rats and effect of administration of melatonin and GH. Data represent mean ± SEM (*n* = 4/5)

Western blot results showed that Bax (*p* ≤ 0.01) and Bad (*p* ≤ 0.005) protein expressions were up regulated during aging (Fig. 7). When old animals were treated with melatonin, a significant reduction of both proteins could be observed (*p* ≤ 0.01). However treatment with GH to old males was only able to reduce Bax protein (*p* ≤ 0.005) in the dentate gyrus (Fig. 7). Aging decreased the expression of Mcl-1 protein (*p* ≤ 0.05) and treatment with melatonin was able to significantly increase this parameter (*p* ≤ 0.001) (Fig. 8). In the case of Bcl-2 protein, results obtained by Western blotting correlated with data obtained by PCR analysis. Bcl-2 protein levels demonstrated a tendency to decrease during aging, and treatments with GH and melatonin were not able to change this parameter (Fig. 9).

**Fig. 10:**
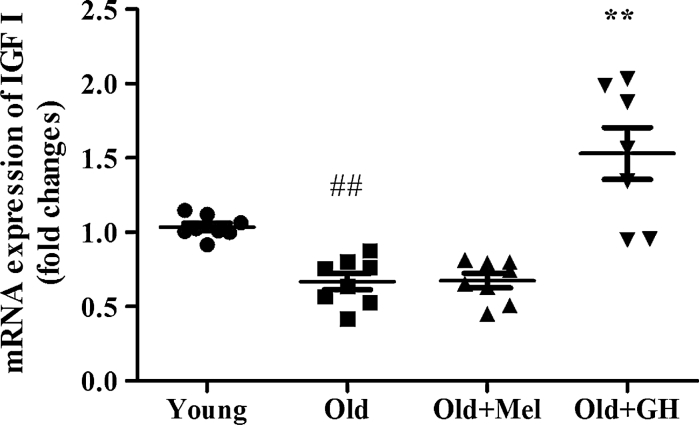
The expression of mRNA IGF1 in the dentate gyrus of young and old male rats and effect of administration of melatonin and GH. Data represent mean ± SEM (*n* = 7/8)

In addition, we also demonstrated that expression of IGFI was decreased in dentate gyrus of old male rats (*p* ≤ 0.02) and treatment with GH was able to significantly increase this parameter (*p* ≤ 0.02) (Fig. 10).

## Discussion

In the present paper, we have demonstrated that aging induced significant increases in gene and protein expressions of pro-apoptotic markers (Bax and Bad) in the dentate gyrus of male rats. Tehranian et al. (2008) found that after cortical contusion injury Bax null mice were able to lose less hippocampal cells and showed increased hippocampal neurogenesis. Results obtained in our research are in accordance with data demonstrating that expressions of Bax and Bid protein in old-aged animals were significantly higher than in the young-control group (Thees et al. 2005; Kim et al. 2010). Our results also demonstrated an increase in the mRNA expression of AIF in the dentate gyrus of old rats and this could suggest, that this brain area was following different apoptotic pathways. Reix et al. (2007) reported that the “apoptotic-inducing” 57 kDa isoform of AIF was increased with age and these authors suggest that the prevalence of AIF-dependent programmed cell death in cerebral cortex was also increased with age.

Another point of our research was the consequence of the expression of proteins and genes with opposite effects on apoptosis. So, we have demonstrated that during aging the expression of Bcl-2 gene and proteins did not change in the dentate gyrus of male Wistar rats. However, the Bcl-2/Bax ratio was significantly higher in the group of young animals as compared with old, indicating a lower level of apoptosis. The Bax/Bcl-2 balance was one of the critical factors determining whether the cells showed undergo apoptosis, and this balance was actually altered during aging (Thees et al. 2005). Interestingly, our present findings indicated that during aging the protein expression of Mcl-1 was decreased, but no changes in the expression of Bcl-2 gene and protein in the dentate gyrus were detected. Yang et al. (2012) demonstrated significant reduction in Mcl-1 expression at both the mRNA and the protein levels and the ratio of expression levels of Mcl-1/Bax genes in the aging subjects. Rapid reduction of Mcl-1 mRNA and protein levels are early events after DNA damage in neurons, and maintaining high Mcl-1 levels can protect neurons against death (Arbour et al. 2008).

The present study demonstrated that aging was able to affect negatively the gene expression of XIAP and NIAP in the dentate gyrus of old male rats. In vitro studies reported that upregulation of NIAP and decreased interaction of NIAP with its endogenous inhibitor, Smac, by the presence of neurotrophin-3, did actually protected neurons from Aβ induced death (Lesné et al. 2005). Trapp et al. (2003) reported that transgenic XIAP-overexpressing mice showed reduced caspase-3 activation and fewer cells with DNA fragmentation.

The brain is exposed to chronic oxidative stress during aging (Rastogi et al. 2012). As result, there is a gradual accumulation of damaged proteins and a functional decline in the brain’s endogenous defense system (Rumora et al. 2007; Min et al. 2008). Our results clearly indicated that levels of HSP 70 were decreased in the dentate gyrus of old male rats. These findings are in consonance with the results obtained by Pardue et al. (1992) and Galli et al. (2006), in which HSP 70 induction and constitutive HSC 70 values are lower with age in hippocampus. Other authors demonstrated that HSP 72 blocked apoptosis primarily by inhibiting translocation of the pro-apoptotic Bcl-2 family member Bax, thereby preventing the release of pro-apoptotic factors from mitochondria (Stankiewicz et al. 2005) and inhibiting AIF translocation to the nucleus (Matsumori et al. 2005).

Our data showed that the expression of SIRT1 was decreased in the dentate gyrus of old rats. A decrease in extracellular signal-regulated kinase 1/2 phosphorylation and altered expression of hippocampal genes involved in synaptic function, lipid metabolism, and myelinization were also detected in SIRT1-KO mice. By contrast, mice with high levels of SIRT1 expression in the brain exhibited normal synaptic plasticity and memory (Michán et al. 2010). Pallàs et al. (2008) have demonstrated in the senescence-accelerated prone mice (SAMP-8), a progressively decreased SIRT1 expression. Lafontaine-Lacasse et al. (2010) have shown that Sirt1 mRNA levels were strongly decreased by aging in the arcuate nucleus. It has been also reported that specific activity of SIRT1 was decreased with aging and this could impact the age-associated protein acetylation levels in the cerebellum (Marton et al. 2010). SIRT2 has been related to synaptic plasticity, learning and memory that are also in turn related to neuronal motility and migration. SIRT2 has also been implicated in neuronal growth cone motility (Harting and Knoll 2010). The data presented here, showed significantly decreased SIRT2 expressions in the dentate gyrus of old male rats.

In our study GH administration was found to increase expression on XIAP, NIAP, SIRT2 and HSP 70 levels and to decrease the expression of pro-apoptotic markers like Bax and AIF. These results demonstrated that GH was able to regulate different (extrinsic and intrinsic) pathways of the apoptotic process in the dentate gyrus of old male rats. The mechanism of regulation of apoptosis by GH could be direct or in-direct, via induction of IGF-1 production and in the literature several results seemed to confirm this. We have demonstrated that the mRNA expression of IGF-1 that showed an age associated decrease in the dentate gyrus in male rats could be restored by GH treatment. Thus restoration of physiological levels of GH/IGF-1 axis was able to influence on the molecular pathways that lead to a reduction in apoptosis. Mechanisms underlying GH-mediated regulation of apoptosis include activation of the phosphatidylinositol 3-kinases (PI3K)-Akt pathway (Jeay et al. 2002), reduction of Bax (Kolle et al. 2002) and increasing Bcl-2 expression (Haeffner et al. 1999). Treatment with GH decreased LDH release, Bax/caspase-3 activity and increased Bcl-2 expression compared with Aβ treatment in a human neuroblastoma cell line (Zhang et al. 2010). Svensson et al. (2008) also found that treatment with GH is capable of preventing or even repairing morphine-induced damage to hippocampal cells by decreasing the caspase-3 activity and increasing the neuronal cell density. Reduction of apoptosis has also been shown by our group to have a positive effect on behavior in old male rats like memory tasks performed in the radial maze and motor ability in the Rotarod treadmill (Esteban et al. 2010). GH improved working memory processes through both NMDA and AMPA glutamatergic receptors and it required the activation of extracellular MEK/ERK signalling pathway. These effects could be related to the enhancement of excitatory synaptic transmission in the hippocampus reported by GH administration (Ramis et al. 2013).

Besides exhibiting well known antioxidant properties, melatonin also has been shown to protect neuronal cells from oxidative stress and apoptosis as induced by mitochondrial DNA deletion (Alvira et al. 2006; Jou et al. 2007). An important observation of the present study is that chronic treatment with melatonin did actually reduce the expression of Bax and Bad in the dentate gyrus of old male rats. These findings together with previous results indicating that melatonin was able to inhibit H_2_O_2_-induced apoptosis in cultured rat astrocytes, point to its ability to down regulate Bax expression and to inhibit caspase-3 activation (Juknat et al. 2005). Baydas et al. (2005) found that chronic melatonin administration markedly reduced the hyperhomocysteinemia-induced rise in mitochondrial Bax levels, restoring cytosolic Bax values and inhibiting caspase-9 activation in the hippocampus. In Tg mice (a model of Alzheimer’s disease) early melatonin supplementation, prevented the abnormal upregulation of Bax, caspase-3 and Par-4 (prostate apoptosis response-4) in cortex neurons (Feng et al. 2006). The other, possible, mechanism of action of melatonin is its upregulation of anti-apoptotic proteins, as has been demonstrated by several authors. So, melatonin increased Bcl-2 and augmented expression of XIAP in ethanol-treated HN2-5 (mouse hippocampal neuron-derived) cells (Shetha et al. 2009). Nopparat et al. (2010) have demonstrated, in the SK-N-SH dopaminergic cell line, a novel role of melatonin in protecting cells from autophagic cell death triggered by the Bcl-2/Beclin 1 pathway, inhibiting the activation of the JNK1 (c-Jun amino-terminal kinase), Bcl-2 upstream pathway. In the present study, melatonin treatment was not able to change the expression of Bcl-2 and XIAP, but significantly increased gene expression of NIAP, Mcl-1 protein levels and could maintain a favourable B-cell lymphoma 2 (Bcl-2)/Bax ratio in the dentate gyrus of old rats. In addition, we have also observed that melatonin was able to decrease mRNA expression of AIF. Our data were in agreement with previous reports in which melatonin was able to prevent the insult related release of AIF from mitochondria (Andrabi et al. 2004). One of the most important changes that we have observed in aged dentate gyrus after melatonin treatment was the increase in the expression of SIRT1, which was consistent with finding by Gutierrez-Cuesta et al. (2008), who examined the effects of melatonin stimulating survival processes in senescence-accelerated (SAMP8) and resistant mice (SAMR1). The same group has also demonstrated that melatonin exerted a neuroprotective role through the SIRT1 pathway (Tajes et al. 2009).

In conclusion, aging in the dentate gyrus is associated with increased expression of pro-apoptotic genes and proteins, like Bax, Bad, AIF and decreased anti-apoptotic ones such as Mcl-1, XIAP and NIAP. We reported also a decrease in expression of SIRT1 and SIRT2 in the dentate gyrus of old male rats. Both treatments have been shown to modulate the pro-antiapoptotic ratio and increase sirtuins expression restoring the situation found in young animals. Our data demonstrated that melatonin and growth hormone could exert a protective effect on hippocampal damage induced by aging. Further studies of the survival signalling pathways may provide novel therapeutic strategies for preventive of age-related disease to prevent age-related alterations.

## Abbreviation

NIAP: Neuronal inhibitor apoptosis protein
XIAP: X-linked inhibitor of apoptosis protein
AIF: Apoptosis-inducing factor
Mcl-1: Myeloid cell leukemia-1
SIRT1 and 2: Sirtuin 1 and 2
HSPs: Heat shock proteins
GH: Growth hormone
Bad: Bcl-2-associated death promoter
Bax: Bcl-2-associated X protein
Bak: Bcl-2 homologous antagonist/killer
Endo G: Endonuclease G
PI3K: Phosphatidylinositol 3-kinases
AKT: Protein kinase B (PKB), is a serine/threonine-specific protein kinase
cAMP: Cyclic adenosine monophosphate
IGF-I: Insulin like growth factor
Bcl-2: B-cell lymphoma 2
CNS: Central nervous system
